# Bilateral oculomotor ocular neuromyotonia: a case report

**DOI:** 10.1186/s12883-018-1142-0

**Published:** 2018-09-03

**Authors:** Tanyatuth Padungkiatsagul, Panitha Jindahra, Anuchit Poonyathalang, Narong Samipak, Kavin Vanikieti

**Affiliations:** 1 Department of Ophthalmology, Faculty of Medicine Ramathibodi Hospital, Mahidol University, 270 Rama VI Road, Bangkok, 10400 Thailand; 2 Department of Medicine, Faculty of Medicine Ramathibodi Hospital, Mahidol University, 270 Rama VI Road, Bangkok, 10400 Thailand; 3 Chakri Nareubodindra Medical Institute, Faculty of Medicine Ramathibodi Hospital, Mahidol University, National Highway 1011, Bang Phli district, Samutprakarn, 10540 Thailand

**Keywords:** Bilateral, Oculomotor, Ocular neuromyotonia, Episodic esotropia

## Abstract

**Background:**

Ocular neuromyotonia (ONM) is characterized by episodic diplopia, which is usually triggered by prolonged eccentric gaze of the affected extraocular muscles. The spell is characterized by involuntary, occasionally painful, sustained contraction of one or more extraocular muscles innervated by the oculomotor, trochlear, or abducens nerve. ONM usually occurs as a late consequence of radiotherapy around the parasellar area, although idiopathic cases have been reported. Most cases are unilateral; however, bilateral ONM has occasionally been described.

**Case presentation:**

A 60-year-old woman presented with a 4-month history of episodic, painful, horizontal binocular diplopia. She underwent external beam radiotherapy to the skull base for treatment of nasopharyngeal carcinoma. The tumor was well controlled. General neurological examination findings were unremarkable. Neuro-ophthalmic examination revealed normal visual acuity, visual fields, pupils, and fundi. Ocular alignment showed orthotropia with normal ocular motility. Myasthenic eyelid signs were absent. However, she developed episodes of involuntary sustained contraction of the medial rectus muscle following prolonged eccentric gaze toward the affected medial rectus muscle, which resulted in esotropia upon returning to the primary position. The esotropic episodes spontaneously resolved after approximately 2 min. These spells affected both medial rectus muscles. Both pupils remained normal throughout the examination. Magnetic resonance imaging revealed neither brain parenchyma/brain stem lesions nor tumor recurrence. Her symptoms were successfully treated with carbamazepine.

**Conclusions:**

Episodic esotropia in the adducting eye following prolonged horizontal eccentric gaze is a significant characteristic of ONM affecting the bilateral medial rectus muscles (i.e., bilateral oculomotor ONM). In spite of its extreme rarity, ONM should be considered as a differential diagnosis of episodic diplopia, especially in patients with a history of radiotherapy around the parasellar area. Careful examination with prolonged eccentric gaze should be performed to achieve a correct diagnosis and avoid an extensive unnecessary workup.

**Electronic supplementary material:**

The online version of this article (10.1186/s12883-018-1142-0) contains supplementary material, which is available to authorized users.

## Background

Ocular neuromyotonia (ONM) is a rare clinical entity characterized by episodic diplopia, which is usually triggered by prolonged eccentric gaze of the affected extraocular muscles (EOMs). The spell shows involuntary, occasionally painful, sustained contraction of one or more EOM innervated by the oculomotor, trochlear, or abducens nerve [1–3]. ONM has been frequently reported as a late consequence of radiotherapy around the parasellar area [3–12]. Some cases have been described in patients with thyroid eye disease [4, 13, 14]. However, idiopathic cases have also been reported [15, 16]. Most cases are unilateral, but bilateral ONM has also been infrequently described [11–13]. We herein report a case of bilateral oculomotor ONM following successful radiotherapy around the parasellar area.

## Case presentation

**Fig. 1:**
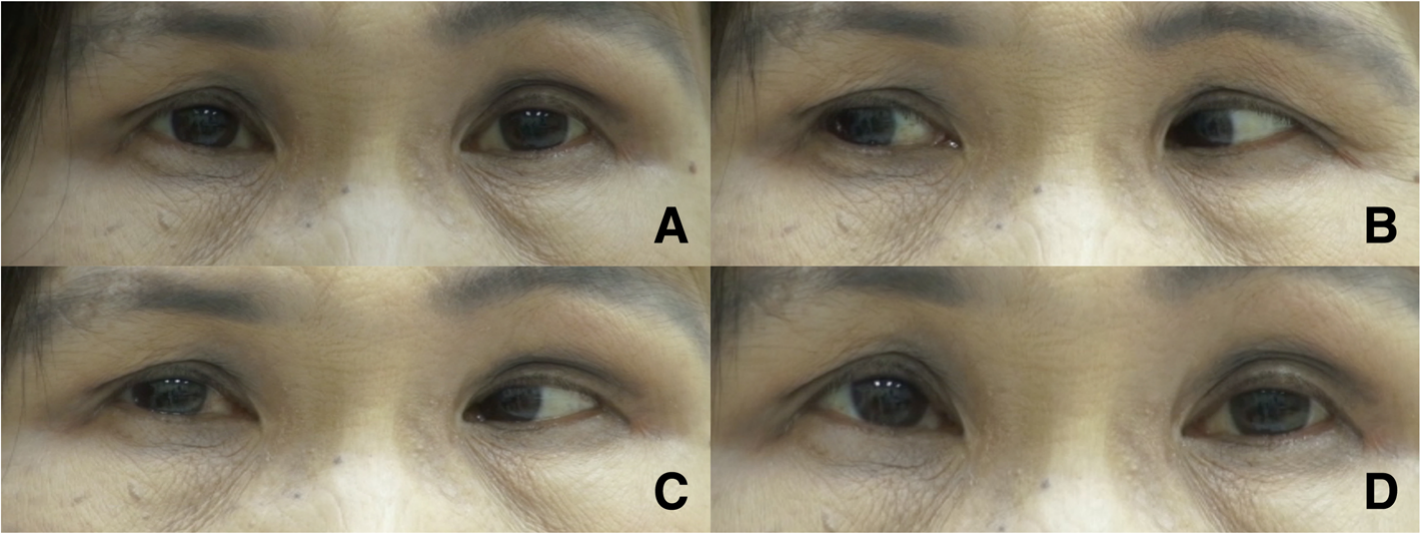
*Left oculomotor ocular neuromyotonia.* The primary position was characterized by orthotropia (**a**). Following 30 s of right eccentric gaze (**b**), the patient developed involuntary contraction of left medial rectus, which resulted in left esotropia while returning both eyes to the primary position (**c**). The left esotropia lasted approximately 2 min, then spontaneously resolved (**d**)

**Fig. 2:**
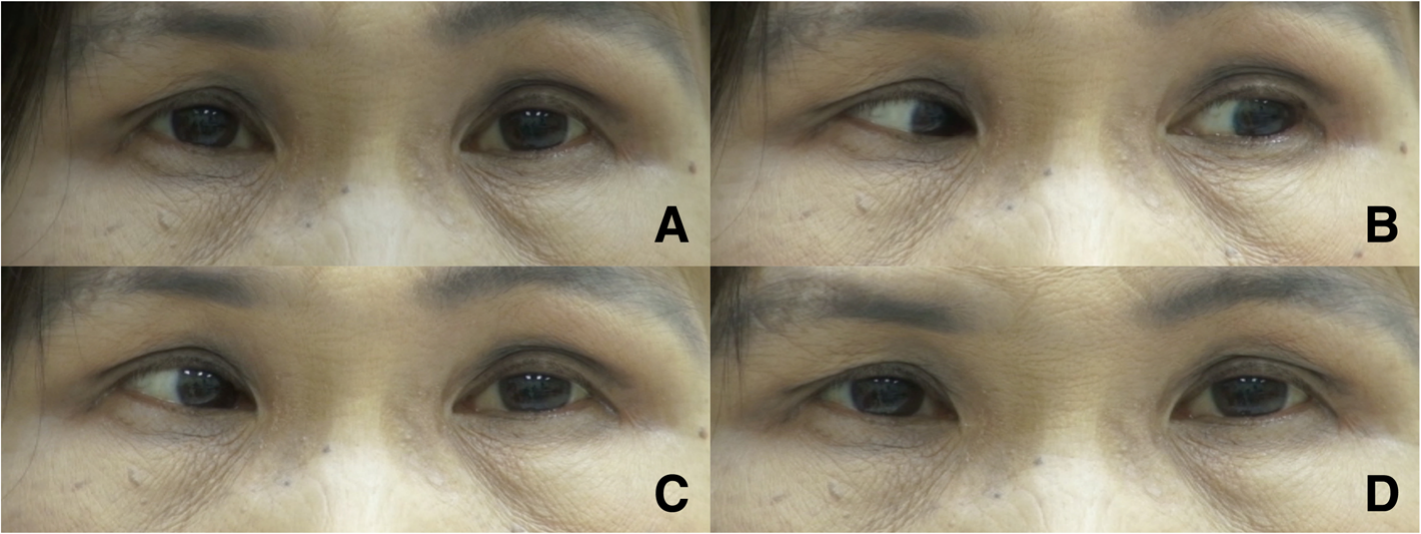
*Right oculomotor ocular neuromyotonia.* The primary position was characterized by orthotropia (**a**). Following 30 s of left eccentric gaze (**b**), the patient developed involuntary contraction of the right medial rectus, which resulted in right esotropia while returning both eyes to the primary position (**c**). The right esotropia lasted approximately 2 min, then spontaneously resolved (**d**)

A 60-year-old Thai woman presented with a 4-month history of episodic, painful, horizontal binocular diplopia. She denied any oscillopsia. She was previously treated for nasopharyngeal carcinoma with several courses of chemotherapy and external beam radiotherapy (total dose of 7000 cGy). The tumor was well controlled; her most recent radiotherapy was administered 13 years previously. General neurological examination findings were unremarkable. Neuro-ophthalmic examination revealed normal visual acuity, visual fields, pupils, and fundi. Ocular alignment showed orthotropia and orthophoria by alternate cover test. Her ocular motility was normal in all gazes, including adduction. Myasthenic eyelid signs, including orbicularis oculi weakness, fatigable ptosis, and Cogan’s lid twitch, were absent. However, following a 30-s right eccentric gaze, she developed involuntary contraction of the left medial rectus, which resulted in left esotropia while returning both eyes to the primary position. The left esotropia lasted approximately 2 min, then spontaneously resolved (Fig. 1 and Additional file 1: Video S1). Likewise, these spells occurred following a 30-s gaze in the opposite direction (left eccentric gaze), which resulted in right esotropia in the primary position (Fig. 2  and Additional file 1: Video S1). During these spells, esotropia was steady (until it waned) and not variable. Both the pupils and the eyelid positions remained normal throughout the examination. No anisocoria was detected during the episodes of esotropia. Prolonged vertical eccentric gaze did not induce any ocular misalignment. Magnetic resonance imaging revealed neither brain parenchyma/brain stem lesions nor tumor recurrence. Her symptoms were successfully treated with carbamazepine at 200 mg daily.

## Discussion and conclusions

The term ONM was first coined in 1970 by Ricker and Mertens [1]. This condition is a rare and unique clinical entity characterized by involuntary episodic diplopia with a duration of seconds to minutes, usually provoked by prolonged eccentric gaze of the affected EOM [1–6]. This condition may involve any EOMs or cranial nerves.

ONM is frequently associated with prior radiation to the skull base [3–12]. However, other causes have also been reported, including thyroid eye disease and carotid artery aneurysm; idiopathic cases have also been described [4, 13–18]. The latency between prior radiation and the onset of diplopia ranges from 2 months to 18 years [19]. The precise pathophysiology is not clearly established. ONM is thought to be caused by segmental cranial nerve demyelination due to radiologic injury or other conditions that compromise the course of neural excitation. Ephaptic neural transmission, which is the transmission of nerve impulses along lateral contact between defective myelinized nerve fibers instead of transmission across a synapse, could explain the intensified prolonged tonic muscle contraction with deficient relaxation of the EOM innervated by the affected nerve [3, 5, 8, 12, 13, 15]. This mechanism is also associated with superior oblique myokymia and facial nerve overactivity [3]. Alternative explanations are axonal hyperexcitability from calcium channel dysfunction or central neural disorganization [3, 20]. Electromyography (EMG) of affected extraocular muscles in quiescent phase may show discharge patterns compatible with neurogenic rather than myogenic disorder [1, 2]. During contracture phase, EMG discloses spontaneous continuous muscle unit activity, with brief bursts of motor unit potentials, firing at high frequency [21]. However, EMG was not performed in our patient given the diagnosis of ONM can be made based on clinical grounds.

Bilateral oculomotor ONM is extremely rare. To the best of our knowledge, this is the second case of bilateral oculomotor ONM ever reported. In 1996, Morrow et al. [12] reported the first case of bilateral oculomotor ONM in a 45-year-old woman who was treated for a pituitary adenoma. Her disease was controlled with surgical resection and radiotherapy to the parasellar region (total dose of 5000 cGy). She developed episodic diplopia involving the bilateral oculomotor nerves innervating the EOMs and levator palpebrae muscles, which resulted in eyelid retraction and EOM limitation of both abduction and vertical movement in the affected eye during the spell [12]. Our patient also had a history of skull base radiation. Although, the latency from the most recent irradiation to the onset of ONM was considerably late, 13 years, the latency have been reported up to 18 years [19]. She developed episodic sustained contraction and delayed relaxation of both medial rectus muscles following prolonged action of the affected EOM, which resulted in a 2-min episode of esotropia while returning both eyes to the primary position. The differential diagnoses of acquired episodic diplopia include convergence spasm, ocular myasthenia gravis, superior oblique myokymia, ocular neuromyotonia, and decompensating heterotropia. In patients with unexplained episodic diplopia, cautious examination should be performed to determine whether pathognomonic clinical characteristics of ONM are present. In our case, the presence of normal pupillary behavior and non-variable esodeviation were not consistent with convergence spasm. Ocular myasthenia gravis was considered less likely given the contraction rather than weakness of the affected EOM while returning both eyes to the primary position following prolonged action of the involved EOM. Myasthenic eyelid signs were also absent. Furthermore, oscillopsia, vertical deviation, and torsional component of superior oblique myokymia were also ruled out in our case. Finally, normal ocular alignment measured by alternate cover test both at distance and near made decompensating heterotropia less likely. According to the proposed pathophysiology, a neural membrane stabilizer such as carbamazepine is often an effective treatment for ONM. Although, some patients can control their attacks by sustaining gaze opposite to the direction that causes symptoms [22]. Our patient was successfully treated with carbamazepine at 200 mg daily.

In summary, bilateral oculomotor ONM is an extremely rare but distinctive condition that can be recognized clinically. In patients with unexplained episodic diplopia, careful examination with prolonged eccentric gaze should be performed to achieve a correct diagnosis and avoid a largely unnecessary workup.

## Additional file

Additional file 1: **Video 1.** *Bilateral oculomotor ocular neuromyotonia*. The video shows episodes of involuntary sustained contraction of the medial rectus muscles following prolonged eccentric gaze toward the affected medial rectus muscles, which resulted in esotropia upon returning to the primary position. The esotropic episodes spontaneously resolved after approximately 2 min. These spells affected the bilateral medial rectus muscles. (MP4 16771 kb)

## Abbreviations

EMG: Electromyography
EOM: Extraocular muscle
ONM: Ocular neuromyotonia
